# Achieving a 25% reduction in premature non-communicable disease mortality: the Swedish population as a cohort study

**DOI:** 10.1186/s12916-015-0313-8

**Published:** 2015-03-30

**Authors:** Ailiana Santosa, Joacim Rocklöv, Ulf Högberg, Peter Byass

**Affiliations:** Department of Public Health and Clinical Medicine, Umeå Centre for Global Health Research, Division of Epidemiology and Global Health, Umeå University, Umeå, 90187 Sweden; Department of Women’s and Children’s Health, Uppsala University, Akademiska Sjukhuset, 75185 Uppsala Sweden; MRC/Wits Rural Public Health and Health Transitions Research Unit, School of Public Health, Faculty of Health Sciences, University of the Witwatersrand, 27 St Andrews Road, Parktown, Johannesburg 2193 South Africa

**Keywords:** 25 × 25 Target, Mortality, Non-communicable disease, Sweden, World Health Organization

## Abstract

**Background:**

The 2012 World Health Assembly set a target for Member States to reduce premature non-communicable disease (NCD) mortality by 25% over the period 2010 to 2025. This reflected concerns about increasing NCD mortality burdens among productive adults globally. This article first considers whether the WHO target of a 25% reduction in the unconditional probability of dying between ages of 30 and 70 from NCDs (cardiovascular diseases, cancer, diabetes, or chronic respiratory diseases) has already taken place in Sweden during an equivalent 15-year period. Secondly, it assesses which population sub-groups have been more or less successful in contributing to overall changes in premature NCD mortality in Sweden.

**Methods:**

A retrospective dynamic cohort database was constructed from Swedish population registers in the Linnaeus database, covering the entire population in the age range 30 to 69 years for the period 1991 to 2006, which was used directly to measure reductions in premature NCD mortality using a life table method as specified by the WHO. Multivariate Poisson regression models were used to assess the contributions of individual background factors to decreases in premature NCD mortality.

**Results:**

A total of 292,320 deaths occurred in the 30 to 69 year age group during the period 1991 to 2006, against 70,768,848 person-years registered. The crude all-cause mortality rate declined from 5.03 to 3.72 per 1,000 person-years, a 26% reduction. Within this, the unconditional probability of dying between the ages of 30 and 70 from NCD causes as defined by the WHO fell by 30.0%. Age was consistently the strongest determinant of NCD mortality. Background determinants of NCD mortality changed significantly over the four time periods 1991–1994, 1995–1998, 1999–2002, and 2003–2006.

**Conclusions:**

Sweden, now at a late stage of epidemiological transition, has already exceeded the 25% premature NCD mortality reduction target during an earlier 15-year period. This should be encouraging news for countries currently implementing premature NCD mortality reduction programmes. Our findings suggest, however, that it may be difficult for Sweden and other late-transition countries to reach the current 25 × 25 target, particularly where substantial premature mortality reductions have already been achieved.

## Background

In the 2012 session of the World Health Assembly (WHA), Member States adopted a resolution calling for a 25% reduction in premature non-communicable disease (NCD) mortality in the period 2010 to 2025 – the so-called “25 × 25” target [1]. Approximately two-thirds of all deaths worldwide are due to NCDs [2], and that is not set to reduce as populations grow older and risks associated with other causes of mortality are controlled. The major issue of concern is the proportion of NCD mortality that occurs in younger age groups. The WHA resolution has been widely misquoted with the omission of the critical word ‘premature’, making the target immediately impossible to achieve in any population where life expectancy is increasing. Where the importance of ‘premature’ mortality has been acknowledged, it has not always been clear how it should be defined [3]. A definition became clear in the 2014 World Health Organization (WHO) Global Status Report [4]: a 25% relative reduction in the “*unconditional probability of dying between ages of 30 and 70 from cardiovascular diseases, cancer, diabetes or chronic respiratory diseases*”. It has also been noted that the 25 × 25 target is intended to apply to all Member States, rather than primarily those from the developing world targeted by the Millennium Development Goals [5].

The apparently simple concept of a common NCD mortality target for all countries, irrespective of disease patterns and health service status, is epidemiologically complex. It is probably not to be expected that there would be universally applicable strategies of equal effectiveness for all situations. Countries varied considerably in terms of stages of epidemiological transition at the 2010 baseline for the 25 × 25 target, from those for which NCD mortality, as yet, accounts for a relatively small proportion of mortality, to those for which premature NCD mortality has been a longstanding issue of concern [6].

Sweden is a country which has reached an advanced stage of epidemiological transition, with high-quality universal health service coverage and high life expectancy. At the start of the WHO 25 × 25 target period in 2010, life expectancy in Sweden was already 83.5 years for women and 79.5 years for men [7]. Whether or not it will be possible for Sweden to achieve the 25 × 25 premature NCD mortality reduction goal set by the international community is, as yet, a matter for conjecture. However, because of the highly functional individual data registers that are routinely maintained in Sweden, it is possible to consider whether an equivalent target might have been achieved in an earlier period. We believe this to be a particularly relevant question, because a number of other countries will be tackling the 25 × 25 premature NCD mortality target at stages of epidemiological transition corresponding to those which have already been encountered in Sweden. There may therefore be lessons to be learnt from the Swedish experience in terms of possible progress elsewhere.

Our aim in this paper is to assess whether a postulated target of a 25% reduction in the unconditional probability of dying between ages of 30 and 70 from cardiovascular diseases, cancer, diabetes, or chronic respiratory diseases during an earlier equivalent time period approximately a generation earlier (1991 to 2006) was achieved in Sweden, using individual national data. Secondary objectives are to assess which population sub-groups were more or less successful in contributing to changes in premature NCD mortality, drawing lessons of global relevance for countries aiming to achieve 25% reductions in premature NCD mortality from 2010 to 2025.

## Methods

The WHO materials specifying the 25 × 25 target do not specify details of the timeframe as clearly as might be expected. Since mortality rates can only be measured for a period of time (for example, over a calendar year) rather than instantaneously, we have taken the view that the endpoints for the 25 × 25 target are mortality rates during 2025. Similarly, if the baseline is 2010, then the starting points have to be mortality rates during 2010, and therefore the overall duration from 2010 to 2025 over which changes are measured can be regarded as the 15-year period between the midpoints of those years. For the earlier period for which Swedish data were analysed, we considered a directly equivalent 15-year period from 1991 to 2006, for which the relevant data were available.

We used retrospective cohort data from Swedish population registers, covering the national population within the age range 30 to 69 years, including records of cause-specific mortality, for the period 1991 to 2006, to construct a dynamic cohort. Individual background characteristics were recorded at the time of entry to the cohort. The data, obtained from the Linnaeus database, were compiled by linking individual national registers from the National Board for Health and Welfare and from Statistics Sweden [8]. The dataset included individual status on socioeconomics, work, family, and residence from Statistics Sweden and sex, age, and cause of death from the National Board for Health and Welfare. Causes of death had been coded according to the International Classification of Diseases 9th and 10th revisions (ICD-9 and ICD-10) based on the ICD codes defined by the WHO [4], classifying NCD deaths into four groups by ICD-10 codes: cardiovascular disease (I00-I99), cancer (C00-C97), diabetes (E10-E14), and chronic respiratory disease (J30-J98). The following potential determinants of mortality were considered: sex (male or female), marital status (partnered, single, or widowed/divorced), educational attainment (primary, lower secondary, upper secondary, tertiary, or unknown), employment status (full-time with high, medium, or low income, part-time, or not employed), and migration status (in-migrant or Swedish-born).

### Statistical analysis

Mortality was analysed in terms of the unconditional probability of dying between ages 30 and 70 from the four NCD groups, calculating unconditional probability using a life table method as specified by the WHO [4]. Mortality rate ratios with 95% confidence intervals (CIs) were calculated from Poisson regression models, using person-years of residence as exposure time. Multivariate Poisson regression analyses were used to assess which population sub-groups were more or less successful in contributing to decreases in premature NCD mortality. The Poisson models included adjustment for age in decades (30–39, 40–49, 50–59, 60–69) and for calendar time in four-year periods (1991–1994, 1995–1998, 1999–2002, 2003–2006). Stata 12 software was used for analyses. Because of the very large size of the dataset, hypothesis testing leading to *P* values was not particularly helpful and comparisons were primarily assessed on the basis of 95% CIs.

## Results

Overall, the mid-year population of Sweden increased from 8,668,066 in 1991 to 9,080,505 in 2006. People aged 30 to 69 years comprised 48.4% of the 1991 population, and increased to 51.6% of the 2006 population, as the population proportion of younger people decreased. A total of 292,320 deaths occurred in the 30 to 69 year age group during the period 1991 to 2006, against 70,768,848 individually registered person-years for the same age range, corresponding to a crude mortality rate of 4.13 per 1,000 person-years. Table 1 shows how this overall mortality was distributed among various cause of death groups and background factors. Out of the total deaths, 215,185 (73.6%) met the WHO definition of premature NCD deaths, comprising cardiovascular (41.8%), cancer (51.7%), diabetes (2.6%), and chronic respiratory diseases (3.9%).

**Table 1:** **Crude mortality rates per 1,000 person-years for the 30 to 69 year age group in the Swedish population during 1991 to 2006, by causes of death and background factors, for 292,320 deaths occurring over 70,768,842 person-years**

	**All causes**	**Non-communicable disease causes**					**Other causes**
		**Cardiovascular**	**Cancer**	**Diabetes**	**Chronic respiratory**	**All**	
**Overall**	4.13	1.27	1.57	0.08	0.12	3.04	1.09
**Sex**							
Female	3.14	0.70	1.57	0.05	0.11	2.44	0.70
Male	5.10	1.84	1.58	0.10	0.12	3.64	1.47
**Age group**							
30–39 years	0.74	0.08	0.15	0.01	0.01	0.26	0.49
40–49 years	1.79	0.36	0.57	0.03	0.02	0.98	0.80
50–59 years	4.49	1.23	1.81	0.08	0.09	3.22	1.27
60–69 years	11.90	4.35	4.75	0.22	0.45	9.78	2.13
**Time period**							
2003–2006	3.76	0.99	1.53	0.08	0.11	2.71	1.04
1999–2002	3.89	1.15	1.51	0.07	0.12	2.86	1.03
1995–1998	4.18	1.34	1.57	0.08	0.12	3.11	1.08
1991–1994	4.75	1.65	1.69	0.08	0.13	3.54	1.21
**Marital status**							
Partnered	3.97	1.23	1.79	0.06	0.10	3.19	0.78
Single	2.98	0.87	0.79	0.07	0.07	1.80	1.18
Widowed/divorced	8.60	2.76	2.97	0.17	0.35	6.24	2.36
**Education level**							
Tertiary	2.38	0.59	1.13	0.03	0.04	1.78	0.60
Upper secondary	3.29	0.93	1.28	0.06	0.08	2.35	0.93
Lower secondary	3.09	0.77	1.05	0.06	0.08	1.96	1.13
Primary	9.55	3.52	3.46	0.20	0.35	7.53	2.02
Unknown	3.93	1.13	1.11	0.08	0.11	2.44	1.49
**Employment status**							
Full-time – high income	3.07	0.92	1.42	0.03	0.04	2.42	0.65
Full-time – mid income	3.08	0.89	1.36	0.04	0.06	2.36	0.72
Full-time – low income	3.41	0.91	1.42	0.06	0.09	2.49	0.92
Part-time employment	2.79	0.67	0.97	0.06	0.06	1.76	1.03
Not employed	10.42	3.80	2.95	0.26	0.44	7.45	2.97
**Migration status**							
In-migrant	3.23	0.92	1.15	0.06	0.08	2.20	1.03
Swedish-born	4.29	1.33	1.65	0.08	0.12	3.19	1.10

**Figure 1:**
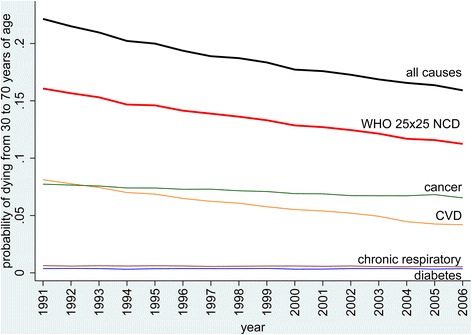
**Overall unconditional probabilities of dying between the ages of 30 and 70 in Sweden from 1991 to 2006, showing non-communicable disease mortality split between cardiovascular, cancer, diabetes and chronic respiratory causes, and all other causes of death.**

From 1991 to 2006, the unconditional probability of death from all causes from 30 to 70 years in Sweden fell by 26.6%. Within this, the unconditional probability of premature death from NCD according to the WHO definition fell by 30.0%. Therefore, the postulated target of a 25% reduction was clearly met. Cardiovascular mortality changes showed the largest reduction (48.3%), as shown in Figure 1. Cancer rates, as the largest of the four components of NCD mortality, declined more modestly (15.5%). Mortality attributed to diabetes accounted for a small proportion of overall mortality and did not contribute appreciably to overall reductions, having reduced by only 1.5%. Chronic respiratory disease was also a minor contributor to overall mortality, but reduced by 19.7%. The rate of reduction in premature NCD mortality declined over the overall 1991 to 2006 period; 54.2% of the overall reduction occurred in the first half of the period.

Since it is clear that many of the factors examined were inter-related – particularly age in relation to marital, educational, and employment status – Table 2 shows an adjusted multivariate Poisson regression model of mortality rate ratios for the same causes of death and background factors as shown in Table 1. After adjustment, age group was the major determinant of NCD mortality.

**Table 2:** **Adjusted mortality rate ratios (95% confidence intervals) by causes of death and background factors, for the 30 to 69 year age group in Sweden during 1991 to 2006, using a Poisson regression model**

	**All causes**	**Non-communicable disease causes**					**Other causes**
		**Cardiovascular**	**Cancer**	**Diabetes**	**Chronic respiratory**	**All**	
**Sex** (reference: Female)							
Male	1.93 (1.91–1.94)	3.18 (3.14–3.23)	1.14 (1.12–1.15)	2.38 (2.25–2.52)	1.38 (1.32–1.44)	1.76 (1.74–1.78)	2.48 (2.44–2.52)
**Age group** (reference: 30–39 years)							
40–49 years	2.99 (2.93–3.05)	5.39 (5.11–5.69)	4.02 (3.86–4.19)	3.61 (3.14–4.16)	3.68 (3.04–4.45)	4.50 (4.30–4.58)	2.31 (2.25–2.37)
50–59 years	7.88 (7.74–8.03)	19.3 (18.3–20.3)	12.8 (12.3–13.3)	9.00 (7.87–10.3)	17.3 (14.5–20.5)	15.1 (14.2–15.1)	4.04 (3.94–4.15)
60–69 years	17.0 (16.7–17.3)	52.5 (49.9–55.3)	29.9 (28.8–31.1)	17.5 (15.3–20.1)	57.8 (48.7–68.6)	37.8 (34.4–36.5)	5.22 (5.08–5.37)
**Time period** (reference: 2003–2006)							
1999–2002	1.06 (1.05–1.07)	1.18 (1.15–1.20)	1.01 (1.00–1.03)	0.94 (0.87–1.01)	1.00 (0.94–1.06)	1.07 (1.06–1.08)	1.04 (1.02–1.07)
1995–1998	1.07 (1.06–1.08)	1.23 (1.21–1.26)	1.02 (1.01–1.04)	0.84 (0.78–0.91)	0.81 (0.76–0.86)	1.09 (1.07–1.10)	1.05 (1.03–1.07)
1991-1994	1.01 (1.00–1.02)	1.17 (1.15–1.20)	0.99 (0.97–1.00)	0.63 (0.58–0.68)	0.61 (0.56–0.64)	1.03 (1.02–1.04)	1.01 (0.99–1.03)
**Marital status** (reference: partnered)							
Single	1.58 (1.57–1.60)	1.68 (1.65–1.70)	1.13 (1.11–1.14)	2.03 (1.90–2.18)	1.88 (1.78–2.00)	1.40 (1.38–1.41)	2.19 (2.15–2.23)
Widowed/divorced	1.68 (1.67–1.70)	1.71 (1.68–1.74)	1.28 (1.26–1.30)	1.90 (1.77–2.03)	2.39 (2.28–2.51)	1.50 (1.48–1.51)	2.45 (2.41–2.50)
**Education level** (reference: tertiary)							
Upper secondary	1.23 (1.21–1.24)	1.37 (1.34–1.40)	1.12 (1.10–1.42)	1.53 (1.38–1.70)	1.71 (1.52–2.08)	1.21 (1.19–1.23)	1.28 (1.25–1.31)
Lower secondary	1.37 (1.35–1.39)	1.50 (1.45–1.55)	1.14 (1.12–1.17)	1.74 (1.53–1.97)	1.99 (1.78–2.22)	1.28 (1.25–1.30)	1.55 (1.50–1.59)
Primary	1.40 (1.38–1.42)	1.68 (1.64–1.72)	1.23 (1.20–1.25)	1.99 (1.79–2.21)	2.11 (1.93–2.32)	1.40 (1.38–1.42)	1.38 (1.34–1.41)
Unknown	1.31 (1.27–1.34)	1.43 (1.36–1.50)	1.21 (1.15–1.26)	1.39 (1.16–1.68)	1.77 (1.52–2.08)	1.26 (1.23–1.30)	1.35 (1.29–1.41)
**Employment status** (reference: full-time – high income)							
Full-time – mid income	1.24 (1.22–1.26)	1.26 (1.23–1.30)	1.16 (1.13–1.19)	1.51 (1.19–1.75)	1.49 (1.31–1.69)	1.23 (1.21–1.26)	1.26 (1.22–1.30)
Full-time – low income	1.80 (1.77–1.83)	1.86 (1.81–1.92)	1.42 (1.38–1.46)	3.25 (2.79–3.78)	2.53 (2.22–2.87)	1.68 (1.65–1.72)	2.21 (2.13–2.28)
Part-time employment	1.73 (1.69–1.78)	1.70 (1.62–1.78)	1.30 (1.25–1.35)	3.08 (2.52–3.76)	2.19 (1.83–2.63)	1.53 (1.48–1.58)	2.35 (2.25–2.46)
Not employed	3.31 (3.26–3.37)	3.88 (3.76–4.00)	1.83 (1.78–1.88)	8.46 (7.28–9.84)	7.42 (6.53–8.43)	2.86 (2.81–2.92)	5.04 (4.87–5.22)
**Migration status** (reference: in-migrant)							
Swedish-born	1.42 (1.40–1.44)	1.49 (1.45–1.52)	1.39 (1.36–1.42)	1.73 (1.58–1.90)	1.87 (1.73–2.02)	1.46 (1.44–1.48)	1.33 (1.30–1.36)

**Figure 2:**
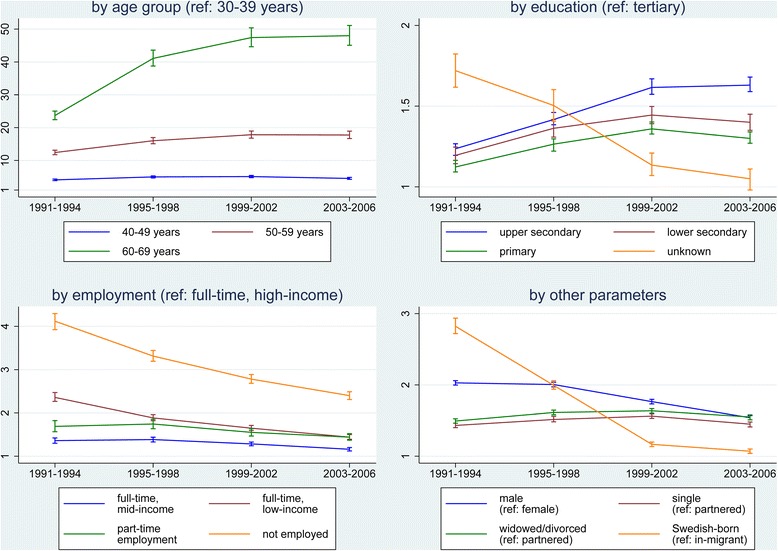
**Adjusted mortality rate ratios and 95% confidence intervals for premature non-communicable disease mortality for the 30 to 69 year age group in Sweden by time period and background factors, using Poisson regression models for each time period.**

Similar adjusted multivariate Poisson regression models were examined for each of the four time periods during the overall 1991–2006 period. Figure 2 shows NCD mortality rate ratios and 95% CIs and background factor, with the same reference categories as in Table 2 (female, 30 to 39 years, partnered, tertiary education, full-time employment with high income, in-migrant). Age group, and to a lesser extent education, became increasingly strong determinants of NCD mortality as time passed. Employment, sex, marital status, and migration status became less strongly associated with NCD mortality as time passed. Excess mortality among males persisted over the four time periods. The in-migrant group experienced a mortality advantage in earlier periods compared with those born in Sweden, but this reduced to a nil difference in the final time period.

## Discussion

Our results show that Sweden has already met the postulated target of a 25% reduction in premature NCD mortality over a 15-year period. We discuss our findings below, firstly, in terms of interpreting the Swedish experience; secondly, examining the implications for the 25 × 25 target in Sweden (and other countries already at a late stage of epidemiological transition by 2010); and thirdly, considering strategic implications that may help countries at earlier stages of epidemiological transition to achieve the 25 × 25 target.

### The Swedish experience of achieving a 25% reduction in premature NCD mortality over 15 years

Although Sweden achieved a 25% reduction in premature NCD mortality during the period 1991 to 2006, this was not done against any specific international target, but simply as part of continuing improvements in public health in Sweden, and against a background of relatively good socio-economic conditions and biomedical advances. Nevertheless, it clearly demonstrates that this rate of reduction in premature NCD mortality in countries at relatively late stages of epidemiological transition is possible.

The unique level of detail and completeness in the Swedish population registers provides an unparalleled opportunity to examine how the 25% reduction in premature NCD mortality came about. Figure 2 shows that there were bigger relative reductions in premature NCD mortality over time for the younger age groups. Mortality rate differences between various sub-groups were very predictable; being partnered, more educated, and employed in higher income groups were all protective against premature NCD death, even though each group underwent its own mortality transition over time. Being male carried a higher risk than most background factors for premature NCD death. These mortality inequalities are generally consistent with those reported from a large-scale European study [9].

The ‘not employed’ and ‘Swedish-born’ categories within the analysis need special interpretation. Those ‘not employed’ included ‘retired’ people (mostly in the 60 to 69 year age group) as well as those who were unemployed for other reasons such as sickness. Consequently, there were relatively high mortality rate ratios associated with not being employed, even after adjustment for age. The in-migrant group experienced a considerable mortality advantage compared with those born in Sweden in earlier periods, but this reduced to a nil difference in the final time period. This may be consistent with a ‘healthy migrant’ effect (whereby self-selection means that migrants might be less likely to have NCDs on arrival), but this difference seemed to diminish over time [10].

Causes of NCDs are multi-factorial, and associated with social determinants of health, including physical, environmental, biological, behavioural, and socio-economic factors. Latency periods between NCD risk exposures and mortality outcomes are also complex. In Sweden, the current prevalence of daily smoking among men is lower than among women and also low compared with other European countries [11]. As smoking has declined significantly in recent decades, alcohol consumption has increased [12]. The proportion of overweight and obese people has also increased, particularly among lower educated groups [13]. Past risk exposures will have driven our mortality findings during the 1991 to 2006 period, and, combined with more recent exposures, will contribute to determining future NCD mortality in Sweden.

We were unable to consider NCD risk factors such as obesity, smoking, alcohol consumption, and physical activity on an individual basis in the Swedish dataset, and hence are unable to conclude on the potential contributions of NCD risk-reduction strategies to the premature NCD mortality reductions observed. Further work is being conducted to examine how major NCD risk factors may have influenced premature NCD mortality reductions in an area of northern Sweden, in a population for which detailed individual risk factor data are available. Modelled findings have suggested that reducing NCD risks can contribute considerably to mortality reductions in the context of the 25 × 25 target [14]. Risk reduction is also, therefore, likely to be an important component of national strategies aimed at the target.

### Implications for the 25 × 25 target in Sweden and other late-transition countries

In real terms for the Swedish population, the reductions in premature NCD mortality achieved from 1991 to 2006 corresponded to about 3,000 premature deaths averted annually. The 25% target reduction was achieved against the background of continuing population ageing in Sweden, though younger age groups benefited to a greater extent from mortality reductions. This suggests that the WHO 25 × 25 target is robustly formulated in considering the unconditional probability of dying, rather than taking any other more complex endpoint. However, having achieved a 25% reduction in a previous generation does not necessarily mean that Sweden can achieve it again for the 2010 to 2025 period, as defined in the WHO 25 × 25 target. It was clear from our analyses that most of the deaths averted in Sweden were associated with reductions in premature cardiovascular mortality, whilst the other major component of premature NCD deaths, cancer mortality, reduced by a much smaller proportion. Furthermore, the overall rate of reduction in premature NCD mortality fell steadily during 1991 to 2006, which does not augur well for what might be achievable in Sweden during 2010 to 2025.

There is therefore a distinct possibility that Sweden, along with other countries already at a late stage of epidemiological transition in 2010, will not go on to achieve the 25 × 25 target, partly because of their earlier successes. In purely demographic terms, there is a potentially perverse effect whereby initial mortality reductions across the 30 to 69 age group may lead to increasingly equal numbers of people in each year of age, hence increasing the proportion of older people within the 30 to 69 age group and so making further mortality reductions less possible. In public health terms, depending what strategies are used to facilitate premature NCD mortality reduction, there may also be diminishing returns over time, after higher-risk individual deaths are initially averted.

Thus, although the WHO 25 × 25 target was aimed at all countries irrespective of their stage of epidemiological transition, it is likely that the outcomes in 2025 will not be independent of countries’ 2010 transitional status. It was also clear from the Swedish case that premature NCD mortality accounted for around three-quarters of all mortality in the 30 to 69 year age group, and so any effective overall mortality reduction in that age group has to be predicated on reducing the NCD component. This is likely to be the case for all countries where external and infectious causes of death have already been relatively well controlled in the 30 to 69 year age group. We suggest that NCD management interventions targeted at the upper range of the premature age group might be the most effective way for countries to reach the 25 × 25 target, although there may be more long-term benefits from NCD risk factor interventions among younger adults.

### Strategic implications for the 25 × 25 target in countries at earlier stages of transition

Unsurprisingly, the overwhelming burden of premature NCD mortality in Sweden occurred among older men, and this is highly likely to follow similar patterns elsewhere. Data from the INDEPTH Network suggested that, for Africa and Asia, where most deaths are not routinely documented, a little under half of adult NCD deaths occurred under 65 years of age, with population-based rates in that age group being broadly similar across Africa and Asia, as well as similar to the rates in Sweden described herein [15]. Since the majority of the world’s population lives in low- and middle-income countries, which are generally at various earlier stages of epidemiological transition than Sweden, it will be important to achieve better measurement and understanding of NCD mortality patterns in such locations in assessing progress towards the 25 × 25 target. Premature NCD mortality in different settings may account for variable proportions of overall mortality, depending on patterns of non-NCD mortality and age-sex population profiles, but there appears to be greater consistency than might be supposed in population-based rates of premature NCD mortality. In Sweden, higher levels of education and income were generally protective factors, and men carried a higher mortality burden, so countries aiming at the 25 × 25 target may well want to develop strategies that target lower socio-economic groups of middle-aged men. The apparently high premature NCD mortality associated with not being employed in Sweden is probably inevitable, and most societies will include sub-groups disadvantaged by life-long medical conditions and other factors that apparently lead to a higher mortality risk. The pattern of mortality in Sweden among in-migrants is interesting in that there was no suggestion from our findings that migrants constituted a group needing special attention in terms of premature NCD mortality risk reduction strategies. This may also hold true elsewhere, because of similar healthy migrant effects.

A global consideration of premature mortality burdens, though necessarily dependent on much scantier data than those available for Sweden, concluded that there were substantial reductions in mortality over the period 1970 to 2010 and was reasonably optimistic about the prospects for further global reductions [16]. This review, however, noted that countries would need to set different priorities depending on existing mortality patterns in order to achieve maximum benefits.

## Conclusions

It is impossible to robustly generalise from the Swedish experience of successful premature NCD mortality reduction, over a period a generation earlier in calendar time than the WHO 25 × 25 target, but nevertheless occurring during a late stage of epidemiological transition. Our findings suggest that it may be difficult for Sweden and other late-transition countries to reach the 25 × 25 target during the current target period, particularly in countries where substantial premature mortality reductions have already been achieved. The Swedish case suggests that, unless there are further major improvements in cancer incidence or survival, it will be hard to reduce premature NCD mortality much further. On the other hand, Sweden has shown that the 25% premature NCD mortality reduction target clearly can be achieved over a 15-year period, which should be encouraging news for countries currently implementing premature NCD mortality reduction programmes. Good civil registration and health information systems as implemented in Sweden, including robust cause of death assignment [17], will be essential to track progress towards the WHO 25 × 25 target in other countries if there are to be clear assessments of premature NCD mortality reductions globally in 2025.

## Abbreviations

ICD: International Classification of Diseases
NCD: non-communicable disease
WHA: World Health Assembly
WHO: World Health Organization

## References

[1] World Health Organization. Sixty-fifth World Health Assembly, second report of Committee A. A65/54. Geneva: WHO; 2012. http://apps.who.int/gb/DGNP/pdf_files/A65_REC1-en.pdf.

[2] World Health Organization. Global status report on non-communicable diseases 2010. Geneva: WHO; 2011. http://whqlibdoc.who.int/publications/2011/9789240686458_eng.pdf.

[3] Beaglehole R, Bonita R, Horton R, Adams C, Alleyne G, Asaria P (2011). Priority actions for the non-communicable disease crisis. Lancet..

[4] World Health Organization. Global Status Report on non-communicable diseases 2014. Geneva: WHO; 2014. http://apps.who.int/iris/bitstream/10665/148114/1/9789241564854_eng.pdf

[5] Beaglehole R, Bonita R, Ezzati M, Alleyne G, Dain K, Kishore SP (2014). NCD Countdown 2025: accountability for the 25 × 25 NCD mortality reduction target. Lancet..

[6] Santosa A, Wall S, Fottrell E, Högberg U, Byass P (2014). The development and experience of epidemiological transition theory over four decades: a systematic review. Global Health Action..

[7] Statistics Sweden. Life expectancy in Sweden 2001–2010. Statistical news from Statistics Sweden, Stockholm: 2011; AM Nr 2011:263.

[8] Malmberg G, Nilsson LG, Weinehall L (2010). Longitudinal data for interdisciplinary ageing research. Design of the Linnaeus Database. Scand J Public Health..

[9] Mackenbach JP, Kulhánová I, Menvielle G, Bopp M, Borrell C, Costa G (2015). Eurothine and EURO-GBD-SE consortiums. Trends in inequalities in premature mortality: a study of 3.2 million deaths in 13 European countries. J Epidemiol Community Health.

[10] Gimeno-Feliu LA, Calderón-Larrañaga A, Diaz E, Poblador-Plou B, Macipe-Costa R, Prados-Torres A (2015). The healthy migrant effect in primary care. Gac Sanit..

[11] Danielsson M, Gilljam H, Hemström O (2012). Tobacco habits and tobacco-related diseases: Health in Sweden: The National Public Health Report 2012. Chapter 10. Scand J Public Health.

[12] Hensing G (2012). The health consequences of alcohol and drug abuse: Health in Sweden: The National Public Health Report 2012. Chapter 11. Scand J Public Health.

[13] Norberg M, Danielsson M (2012). Overweight, cardiovascular diseases and diabetes: Health in Sweden: The National Public Health Report 2012. Chapter 7. Scand J Public Health.

[14] Kontis V, Mathers CD, Rehm J, Stevens GA, Shield KD, Bonita R (2014). Contribution of six risk factors to achieving the 25x25 non-communicable disease mortality reduction target: a modelling study. Lancet..

[15] Streatfield PK, Khan WA, Bhuiya A, Hanifi SM, Alam N, Bagagnan CH (2014). Adult non-communicable disease mortality in Africa and Asia: evidence from INDEPTH Health and Demographic Surveillance System sites. Global Health Action..

[16] Norheim OF, Jha P, Admasu K, Godal T, Hum RJ, Kruk ME (2015). Avoiding 40% of the premature deaths in each country, 2010–30: review of national mortality trends to help quantify the UN Sustainable Development Goal for health. Lancet..

[17] Sankoh O, Byass P (2014). Time for civil registration with verbal autopsy. Lancet Global Health..

